# Evolutionary Dynamics Do Not Motivate a Single-Mutant Theory of Human Language

**DOI:** 10.1038/s41598-019-57235-8

**Published:** 2020-01-16

**Authors:** Bart de Boer, Bill Thompson, Andrea Ravignani, Cedric Boeckx

**Affiliations:** 1 AI-lab, Vrije Universiteit Brussel, Pleinlaan 2, 1050 Brussel, Belgium; 2 Language and Cognition Department, Max Planck Institut für Psycholinguistik, Wundtlaan 1, 6525 XD Nijmegen, The Netherlands; 3 Research Department, Sealcentre Pieterburen, Hoofdstraat 94a, 9968 AG Pieterburen, The Netherlands; 4 ICREA, Passeig Lluís Companys 23, 08010 Barcelona, Spain; 5 Institute of Complex Systems (UBICS), Universitat de Barcelona, 585 Gran Via, 08007 Barcelona, Spain; 6 Section of General Linguistics, Universitat de Barcelona, 585 Gran Via, 08007 Barcelona, Spain

**Keywords:** Genetic models, Evolution of language, Evolutionary biology

## Abstract

One of the most controversial hypotheses in cognitive science is the Chomskyan evolutionary conjecture that language arose instantaneously in humans through a single mutation. Here we analyze the evolutionary dynamics implied by this hypothesis, which has never been formalized before. The hypothesis supposes the emergence and fixation of a single mutant (capable of the syntactic operation *Merge*) during a narrow historical window as a result of frequency-independent selection under a huge fitness advantage in a population of an effective size no larger than ~15 000 individuals. We examine this proposal by combining diffusion analysis and extreme value theory to derive a probabilistic formulation of its dynamics. We find that although a macro-mutation is much more likely to go to fixation if it occurs, it is much more unlikely *a priori* than multiple mutations with smaller fitness effects. The most likely scenario is therefore one where a medium number of mutations with medium fitness effects accumulate. This precise analysis of the probability of mutations occurring and going to fixation has not been done previously in the context of the evolution of language. Our results cast doubt on any suggestion that evolutionary reasoning provides an independent rationale for a single-mutant theory of language.

## Introduction

## The Chomskyan Evolutionary Conjecture

For centuries, countless scholars have viewed the human capacity for language as central to our species’ unique form of intelligence. Understanding the emergence of this capacity is a core challenge for the cognitive and biological sciences. One of the most influential theories of the human language faculty, articulated over decades by Chomsky and associates^1,2^, proposes that (modern) humans are genetically-equipped with a unique computational capacity that specifically allows us to implement computations over hierarchically structured symbolic representations. According to the more recent formulation of this hypothesis, this capacity is enabled by a single syntactic operation known as *Merge*, which is the basis of our ability to represent complex grammars in a way that other species cannot^2^. Put simply, Merge takes two linguistic units (say, words) and combines them into a set that can then be combined further with other linguistic units, effectively creating unbounded linguistic expressions. These, in turn, are claimed to form the basis for our cognitive creativity and flexibility, setting us aside from other species.

The strongest version of Chomsky’s hypothesis suggests that the biological foundation of Merge is a single genetic mutation. Given its staggering consequences, a mutation of this kind is considered a *macro-mutation*. This controversial hypothesis leads to the conclusion that our modern language capacity emerged instantaneously in a single hominin individual who is an ancestor of all (modern) humans. The conclusion follows from a theory-internal hypothesis that *Merge* is either present in full or totally absent^3^ (Martins and Boeckx present a critical discussion^4^).

The most detailed articulation of the evolutionary conjecture we will examine here has been presented in Berwick and Chomsky^3^. These authors argue that evolutionary considerations represent an independent motivation for the single-mutant theory, because the narrow historical time-window in which the human language faculty is said to have emerged rules out the emergence and fixation of more than one language-relevant genetic anomaly. This evolutionary motivation for a minimalist view of grammar (essentially, the single addition of Merge) is important because it represents a theory-external rationale for the hypothesis. In this paper, we examine this evolutionary proposal formally by conducting a probabilistic analysis of the evolutionary dynamics that result from its assumptions. Specifically, we formalize the hypothesis that fixation of multiple interacting mutations is less probable than fixation of a macro mutation in this time window, and show that this hypothesis is wrong.

The key details of the evolutionary conjecture, presented concisely in Berwick and Chomsky^3^, are as follows: (1) “universal grammar” (a hypothesized innate basis for the computational capacities implied by their theoretical framework of language) can be pared down to little more than a basic combinatorial property known as Merge – this is the major conclusion of the so called ‘minimalist program’^2^; (2) only (modern) humans have Merge; (3) Merge is the result of a single mutation, perhaps provoking a rewiring in the brain yielding novel neural circuitry or network configuration that is missing in other species, and this only arose once in our lineage; (4) because it rests on a single mutation, Merge (as a formalization of a core aspect of language) has the additional virtue that its genetic basis could be very recent in evolutionary terms, in the sense that Merge has no external prerequisites and therefore the time window between no-Merge and Merge can be small, and this is not true of alternative hypotheses; (5) finally, Chomsky and associates insist that this mutation be kept separate from any considerations of communication: according to their arguments, language is primarily advantageous as a means for internal thought and this is the only phenotypic consequence relevant to the fitness of a hypothetical single-mutant (Chomsky and colleagues take other aspects of language, such as the neural control over the vocal apparatus to produce speech, to be ‘peripheral’.)

Berwick and Chomsky’s account, to date the most detailed articulation of the evolutionary scenario in the mainstream generative research tradition, has been criticized on a variety of grounds^5,6^. Although we generally agree with the thurst of these criticisms that dispute arguments 1 through 5, our article does not engage with them. Instead, for the purposes of our analysis, we grant all of Berwick and Chomsky’s assumptions in full, and confine ourselves to examining the consequent evolutionary dynamics formally. In particular, we examine the dynamics of a single, critical, mutation spreading rapidly through a population in a given time window under frequency-independent selection. It is this claim that allows Berwick and Chomsky to argue for a significant selective advantage for Merge–one that places language at the center of a ‘great leap forward’ view of the human cognitive revolution advocated by Klein^7^.

Our evolutionary analysis is made possible by two insights. First, Berwick and Chomsky’s hypothesis is sufficiently precise to place approximate bounds on ordinarily under-determined variables of an appropriate evolutionary analysis, specifically with respect to population size, time-window, and selection regime: these unknown factors can be approximated by combining Berwick and Chomsky’s theoretical proposals with contemporary genetic and demographic findings.

**Figure 1:**
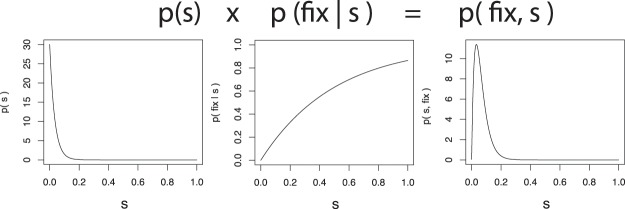
Probabilistic perspective on the Berwick-Chomsky conjecture. From left to right: the a priori probability density of a mutation of a given fitness advantage occurring, the conditional probability density of a given mutation going to fixation (starting with a single mutant) and the total probability density of a mutation occurring and going to fixation.

Second, the appropriate degree of belief in Berwick and Chomsky’s proposal has a natural formulation in probabilistic terms, in the sense that it should factor into two distinct quantities: the a priori probability of a mutation conferring fitness effects commensurate with their hypothesis of Merge; and the conditional probability that this mutation would lead to the scenario we observe today – universal acquisition of language among human populations, or *fixation* in the relevant time window. This interpretation enables an examination via separation of the total probability of an evolutionary scenario into: 1) the probability of mutations arising; and 2) the probability of mutations going to fixation (Fig. 1). Both of these quantities are well-studied evolutionary phenomena, amenable to approximate analysis. The a priori probability of a mutation can be expressed using results from extreme value theory^8^; the probability of today’s scenario conditional on the Merge-mutant hypothesis can be understood as approximately quantifiable through analysis of *fixation* probabilities: the probability that a single Merge mutant goes to fixation in human populations. Fixation probabilities can be calculated in finite populations via numerous standard methods; here we use diffusion analysis^9,10^.

Our analysis contrasts Berwick and Chomsky’s proposal with a scenario in which genetic bases of our linguistic ability evolved through a gradual accumulation of smaller biological changes. This scenario can be articulated in many different ways, for instance as syntax evolving from phonological form^11^, from rapid manual actions^12^ or from much simple pragmatic sequencing of words^13^ (for a comprehensive range of options, see for instance the Oxford Handbook of Language Evolution^14^, in particular part IV). However, nothing we discuss here requires us to choose a specific formulation of this general gradual scenario.

## Model: A Probabilistic Evolutionary Analysis

### The probability of one big step versus many smaller steps

According to the single-mutant view, no other species, not even our closest extinct relatives the Neanderthals, ever had Merge, and Merge was universally in place before *Homo sapiens* moved out of Africa, restricting its emergence to a time window of approximately 100 000 years. Independent evidence indicates that the effective population size of ancient human populations during this period was maximally around 10 000–15 000 individuals with a considerably smaller population bottleneck (hundreds of effective individuals) around 140 000 years ago^15^. Small effective population sizes are supported by observations of ethno-linguistic group sizes of modern hunter-gatherers, which tend to be around 1500^16^. Although there may be many of such groups, thus resulting in a larger number of individuals (the “census size”) because most reproduction occurs within the groups, the effective population size tends to be much smaller than the census size, and closer to the group size.

Our analysis of Berwick and Chomsky’s proposal examines the fate of a single mutant that arises in a population of this size. Conveniently, fixation of a single mutant in a small finite population is an extensively studied dynamics: a  range of theoretical tools exists (which we will present in sections 2.2 and 2.3 below) for analysis of the core proposal. The single mutation envisaged must have conferred an enormous fitness advantage. Formally, fitness is usually expressed as a *selection coefficient*. Only the ratio of fitness of different individuals is generally relevant, so the wild type (i.e. the individuals without mutations) is defined to have fitness 1. Mutant individuals have fitness 1 + *s*, where *s* is called the *selection coefficient*. Because in many cases (as in the case at issue) the organisms under consideration have two copies of each gene, there will be two types of mutant individuals: heterozygous (one copy of the mutation) and homozygous (two copies of the mutation). These in general have different selection coefficients: *s*_*aA*_ and *s*_*AA*_ respectively in our notation.

Selection is usually assumed to be weak in evolutionary models, which licenses many useful methods of approximate analysis. These methods are unavailable to us as a result of the uncommon assumption that a single mutation confers a large fitness effect. This effect cannot be arbitrarily large, because the number of offspring that an individual could raise in prehistoric and pre-agricultural times was limited by many unrelated factors. One way to balance these constraints is to limit our numerical analyses to selection coefficients *s* ≤ 1 (which still corresponds to mutants having on average twice the number of offspring than the wild type in the strongest case). Berwick and Chomsky’s assumption that fitness advantages reflect increased capacity for individual thought rather than communication implies frequency-independent selection.

**Figure 2:**
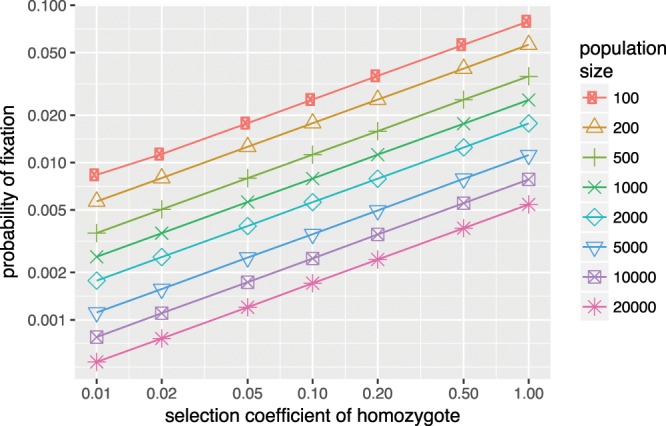
Fixation probabilities for recessive beneficial mutations. Note that these are very low given the large selection coefficients.

Finally, the fitness effect of the proposed mutation would occur in the heterozygote mutant, and its effect must be fully dominant. In other words, both individuals with one copy (heterozygotes) and with two copies (homozygotes) of the mutant gene would have full Merge and thus the full fitness advantage. This is because in the proposed scenario, one either has Merge or not, and therefore there cannot be two separate stages with different fitness effects. The alternative of a recessive mutation, in which individuals need two copies of the mutated gene to have merge, would lead to a strongly reduced probability of the mutation spreading, because homozygotes would be rare initially and the mutant gene would therefore first have to spread through random drift. Figure 2 provides a sample of probabilities; for an exact description of these calculations see the next subsection.

By contrast, the alternative scenario for gradual evolution of linguistic ability proposes that the evolution of language happened in a way that is far less exceptional by biological standards. This means that there were potentially many mutations involved that had small to moderate fitness effects and that all contributed to the ability for language. Because of the smaller fitness effects, fixation probabilities were lower and fixation times were longer. This does not mean that the overall probability of this scenario is lower, however: multiple mutations evolving in parallel can be present in a population at the same time. If the fitness effects are sufficiently small and additive, mutations can be considered to evolve independently (for a discussion of why we think this is justified, see Supplementary Material S7). Also, because the effects of the mutations are small, it is much more probable that if one mutation disappears from the population, eventually another mutation with a similar effect occurs. Finally, in this scenario it is not necessary that all mutations reach full fixation: it is expected that there is some remaining variation in the population. However, the effect of this variation may be small, as there are many genes involved and the variation for a trait determined by many genes with additive effects decreases with the number of genes involved. Unlike the single-mutant hypothesis, there is no reason in this case to assume that the mutations involved were purely dominant. It is unlikely that they were recessive, because recessive mutations of any size are unlikely to spread. However, they may have been semidominant, in which homozygotes have a higher fitness than heterozygotes, which results in higher fixation probabilities and somewhat lower fixation times.

The mutations involved in the gradual scenario may have had positive frequency dependence, because this hypothesis, unlike the Chomskyan view, does not rule out a selective advantage conferred by communication. Positive frequency dependence leads to lower fixation probabilities and longer fixation times, as initially the mutation may not have an influence on fitness. This is quantitatively investigated in section 2.2.4.

### Fixation probability and time

Our analysis makes use of the diffusion method developed by Kimura^9,10^. This approach models the proportion of mutants in a population as a continuous value (in reality the proportion is discrete, because the population consists of a finite number of discrete individuals) and it models evolution of the number of mutants as a diffusion process. This approach is flexible enough to model individuals as haploid or diploid, and in the case of diploid individuals it can model different fitness values for heterozygous and homozygous mutants.

#### General approach

The diffusion approach only considers the evolution of the proportion *p* of mutant alleles *A*, with the wild-type allele denoted as *a*. Following Kimura^17^, the evolution of a finite population of diploid individuals can be approximately described by a partial differential equation:

1 $$\frac{\partial u(p,t)}{\partial t}={M}_{\delta p}(p)\frac{\partial u(p,t)}{\partial p}+\frac{1}{2}{V}_{\delta p}(p)\frac{{\partial }^{2}u(p,t)}{\partial {p}^{2}}$$

(the Fokker-Planck equation, see Supplement S0 for details) featuring the probability *u*(*p,t*) of the mutant allele becoming fixed at time *t*, the mean change ${M}_{\delta p}(p)$ in proportion *p* per generation, and its variance ${V}_{\delta p}(p)$. Assuming that there is no more mutation after the initial mutation that generates mutant allele *A*, the mutants will either disappear or take over the population with probability $u(p,\infty )$_._ (depending on the boundary conditions, this can represent either) and as time tends towards large values, the derivative to time of *u* tends to zero. We can then impose these conditions of disappearance or takeover in the partial differential equation (see Supplement S0). To find a solution to the partial differential equation, we need approximations to the mean change ${M}_{\delta p}(p)$ in proportion *p* per generation, and its variance ${V}_{\delta p}(p)$.

#### Mean and variance terms

There are different ways of approximating the mean and variance for a given population, and these result in different expressions for ${M}_{\delta p}(p)$ and ${V}_{\delta p}(p)$. The simplest way is to model the population as if it were haploid (see Supplementary Section S1 for a demonstration that this way is also the one that most closely approximates Monte Carlo simulations). The reason that this works even for diploid organisms is that initially, homozygous mutants will be very rare (in a randomly mixing population of sufficient size). Therefore, the mutant allele may become so frequent before homozygotes play a role in its evolution, that it is highly likely that the mutant allele reaches fixation. We can therefore ignore the existence of heterozygotes. The mean change and its variance (see Supplementary Section S2 for a derivation) are then:

2 $${M}_{\delta p}(p)=p(1-p)\frac{{s}_{aA}}{1+{s}_{aA}\cdot p}$$

3 $${V}_{\delta p}(p)=\frac{p(1-p)}{2N}\frac{2+{s}_{aA}}{1+{s}_{aA}\cdot p}$$

where *s*_*aA*_ is the selection coefficient of the heterozygote mutant (i.e. assuming the fitness of the wild type is 1, the fitness of the heterozygote mutant is 1 + *s*_*aA*_) and *N* is the population size. Assuming a haploid population is mathematically equivalent to assuming a diploid population with *s*_*AA*_ = 2*s*_*aA*_, (this is also called the case of no dominance or semidominance).

This simple approximation also yields a closed form solution for the fixation probability described by Eq. (1):

4 $${p}_{fix}({p}_{0})=u({p}_{0},\infty )=\frac{1-{e}^{-4N\frac{{s}_{aA}}{2+{s}_{aA}}{p}_{0}}}{1-{e}^{-4N\frac{{s}_{aA}}{2+{s}_{aA}}}}$$

where *p*_0_ is the initial frequency of mutant alleles (which is 1/2 *N* when starting with a single heterozygous mutant).

#### Fixation time

In order to estimate the time it takes for a mutant to reach fixation in a population (starting with a given proportion of mutants) the Fokker-Planck Eq. (1) can be adapted. Setting it equal to minus the fixation probability (instead of 0) results in an equation for the conditional fixation time $\vartheta (p)$, i.e. the product of the probability of reaching fixation multiplied by the time needed to reach fixation, starting with a proportion *p* of mutants (using equations XII.3.4a and XII.3.4b from van Kampen^18^):

5 $$-{p}_{fix}(p)={M}_{\delta p}(p)\frac{\partial \vartheta (p,t)}{\partial p}+\frac{1}{2}{V}_{\delta p}(p)\frac{{\partial }^{2}\vartheta (p,t)}{\partial {p}^{2}}$$

and using boundary conditions:

6 $$\vartheta (0)=\vartheta (1)=0$$

The unconditional fixation time $\tau (p)$ can then be calculated by dividing the conditional fixation time by the fixation probability. Although ignoring the fact that the population consists of heterozygotes may work well for calculating fixation probabilities, it is not expected to work well for calculating fixation times. This is because it is assumed in our analysis that homozygote mutants have no advantage over heterozygote mutants, while approximating the population as haploid implicitly assumes that homozygotes have a selection coefficient that is twice that of heterozygotes. For this reason Kimura’s^17^ approximation is used:

7 $${M}_{\delta p}(p)=p(1-p)({s}_{aA}+({s}_{AA}-2{s}_{aA})p)$$

8 $${V}_{\delta p}(p)=\frac{p(1-p)}{2{N}_{e}}$$

where *N*_*e*_ is the *effective population size*, i.e. the size of a randomly mating population that behaves the same as the actual population (which may not have random mating). These appear to give satisfactory values compared to the Monte-Carlo simulation (see Supplementary Section S1).

**Figure 3:**
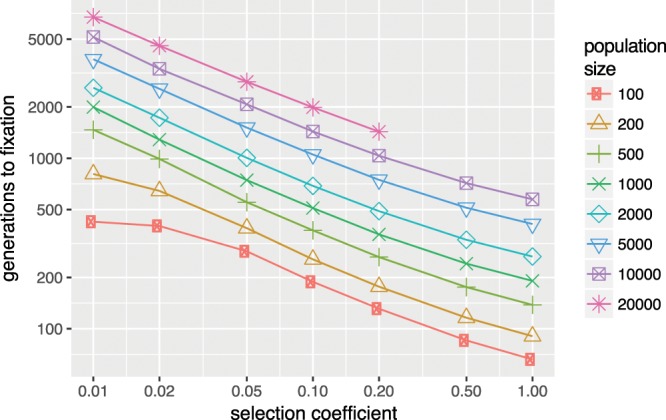
Unconditional mean fixation times for different selection coefficients and population sizes (in individuals, so the number of alleles is twice the number). Note that the missing points in the graph could not be calculated because of numerical limits. Generations can be converted in years by multiplying with generation time, which is from 25–30 years (20).

Solving the resulting equations numerically, we obtain fixation times, expressed in generations shown in Fig. 3. It shows that for small values of *N*∙*s*, fixation time is approximately 4 *N*, which corresponds to the theoretical value for drift^19^. For larger values, fixation time drops rapidly.

#### Frequency dependent fitness

When fitness is (positively) frequency dependent, fixation probabilities are expected to become smaller, as initially the spread of mutants will only be due to drift; if the mutation is rare, individuals that have it will have little or no advantage of it. A simple model for frequency dependent fitness is to make the fitness of a mutant individual linearly dependent on the frequency *p* of mutants in the population, with one linear coefficient for the heterozygote and another for the homozygote (see Supplement S3 for details). These frequency dependent selection coefficients can be substituted in the equations for the mean change and the variance in (2) and (3) and the fixation probabilities (and fixation times) can be estimated.

**Figure 4:**
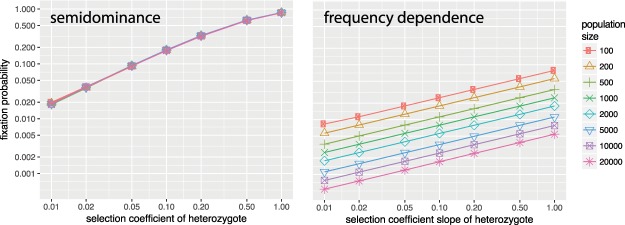
Comparison of the fixation probabilities of the semidominant case and the case where fitness has positive (linear) frequency dependence. To be as close as possible to the semidominant case, the slope of homozygote fixation is set as twice as that of the heterozygote fixation. Note that the vertical axis (of fixation probabilities) is logarithmic, and that therefore the fixation probabilities in the frequency dependent case are much lower than in the semidominant case.

As can be seen in Fig. 4 the fixation probabilities become dramatically lower in the case of frequency dependent selection, even for high values of the slope of the selection coefficients (implying high values of the selection coefficients when mutant frequencies become sufficiently high).

#### Time and probability conclusion

Fixation probability can be modeled with satisfactory accuracy (given that our model parameters are approximations anyway) by using the haploid/semidominant approximation. Fixation time does need to take into account whether the mutation under investigation is dominant, recessive or otherwise. However, assuming the proposed mutation for Merge was dominant and conveyed a large fitness advantage, neither fixation probability nor fixation time are obstacles to the hypothesis. The many mutations with small fitness effects proposed by the gradual scenario would take longer to reach fixation and would be less likely to do so. However, as we already remarked above, multiple mutations of this kind could spread at the same time, and importantly, it is less problematic for this scenario if one mutation does not reach fixation, because it is expected that similar mutations will recur, while the proposed macromutation for Merge will not.

To revisit the probabilistic interpretation we laid out earlier, an appropriate summary of the preceding findings is that as far as fixation probabilities go, both hypotheses (single-mutant and gradual evolution) assign a realistically high conditional probability to the observation that language is universal in our species (i.e. the mutations have spread to fixation or near fixation), each for different reasons. In other words, consideration of fixation dynamics does not decisively distinguish between these two competing hypotheses; both are plausible routes to fixation in populations of approximately the hypothesized size and time depth, conditional on the emergence of the relevant mutation(s) in the first place. As a result, evolutionary support for these hypotheses turns exclusively on consideration of their *a priori* probability.

### Probability of mutations

Orr offers an appropriate theoretical result for the distribution of the size of beneficial mutations, based on extreme value theory^8^, the theory that considers the distribution of the extreme values of a large random sample. Orr assumes that there are a potentially large number of variants of the genome, all with fitness effects that have been drawn from a distribution that fulfills reasonable assumptions. More specifically, the distribution has to be of Gumbel type 1 (or exponential type), a class of distributions that encompasses most everyday distributions with infinite support, such as the Normal (and log Normal) distributions, the Weibul distribution and the exponential distribution. He further assumes that the wild type already has relatively high fitness compared to all possible variants, because it already is the result of many generations of selection (in Orr’s view fitness is already high because organisms are already highly complex, but it is also compatible with Berwick and Chomsky’s view that the mutation for Merge occurred in creatures that already had a fairly rich cognitive repertoire). There are therefore only comparatively few variants that would result in higher fitness. In this case it can be shown (using extreme value theory) that the fitness of beneficial mutants follows an exponential distribution, and that the parameter of this distribution is equal to the reciprocal of the expected value of the fitness difference between the best and second best mutants. This leads to the following expression for the probability *p* of a fitness effect:

9 $$p(s)=\frac{1}{E[{\Delta }_{1}]}{e}^{-\frac{1}{E[{\Delta }_{1}]}s}$$

where *s* is the increase in fitness, which is equivalent to the selection coefficient used in section 2.2 and $E[{\Delta }_{1}]$ the expected value of the difference in fitness between the best and the second best mutations. Experimental evidence is in general agreement with the predictions implied by this model^20^ (but see Supplement S4 for a detailed discussion of its pros and cons).

**Figure 5:**
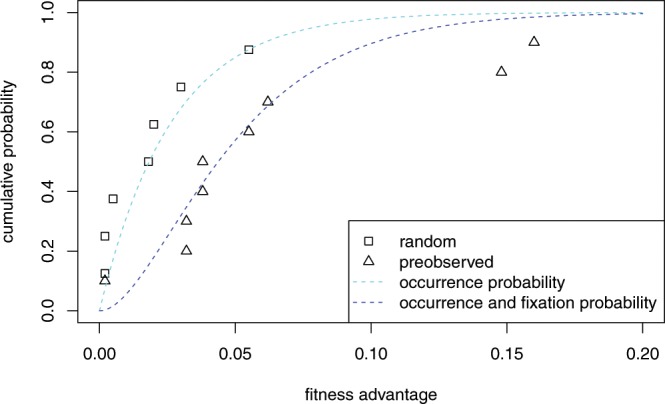
Fitness advantage of viruses from Sanjuán *et al*.‘s (23) sample. The interpolated curve (minimal sum of squares fit) on the random data points (the light blue dashed line and the squares) results in a value of E[Δ_1_] of 1/37. Based on this value, the theoretically predicted curve (the dark blue dashed line and the triangles) for the preobserved mutations (occurrence and going to fixation) fits the data well. For these curves a large effective population size was assumed, so the term with N in Eq. (10) was ignored.

In order to use the exponential distribution to estimate the probability of an evolutionary scenario, it is necessary to have an estimate of the parameter *Δ*_1_. For this it is necessary to have data about the fitness effects of beneficial mutations, and in particular data about the distribution of such mutations when they occur, as opposed to after they have gone to fixation (which would conflate their probability of occurrence with the probability of going to fixation). For practical reasons, data of this kind are hard to obtain, and only exist for very rapidly evolving organisms such as viruses. A small data set is presented in Sanjuán *et al*.^21^ Fitting an exponential distribution to their datapoints of “random” mutations, which are the ones needed to estimate Δ_1_, a value of $E[{\Delta }_{1}]$ of between 1/53 (for maximum likelihood estimation) to 1/37 (for least sum of squares estimation) is found. The data, the interpolation and the theoretical prediction are given in Fig. 5.

Given the small sample, and given that it is unknown how well the data for viruses map on data for humans, there is considerable uncertainty in this estimate. Because lower values correspond to lower probability of large mutations, and in order to prevent an unjustified bias against large mutations, an estimate of 1/30 for the parameter of the exponential distribution is a reasonable approximation.

### Probability of the two scenarios

In order to compare the two scenarios, it is necessary to calculate the probability of a single mutation occurring and going to fixation. For clarity’s sake the term *improvement* is introduced for the event of a beneficial mutation occurring *and* going to fixation. The magnitude of the improvement is defined to be equal to the fitness advantage of the mutation. Finally, the probabilities of single improvements must be combined in order to calculate how many improvements are needed in order to obtain the desired trait.

#### The probability of a single improvement

The probability of a single improvement is the product of a mutation of a given fitness advantage occurring Eq. (9), and that mutation going to fixation. It has been shown that Eq. (4) gives a satisfactory approximation of the latter probability. Unfortunately, as far as we know there is no closed-form solution for the integral of the product of (6) and (12) (which would give the cumulative distribution function). However, there is a very good closed-form approximation (see Supplementary Material S5), resulting in the following cumulative distribution function of a single improvement:

10 $${\rm{CDF}}(s)=1-\frac{{e}^{-\alpha s}}{\beta }[\frac{1}{\alpha }-\frac{1}{2+\alpha }{e}^{-2s}+\frac{1}{N(N+\alpha +1)}{e}^{-(N+1)s}]$$

where and *β* is a scaling factor. That this equation is a good approximation is illustrated by the fit to the experimental observations illustrated with the dark blue line in Fig. 5.

#### The expected number of improvements necessary

**Figure 6:**
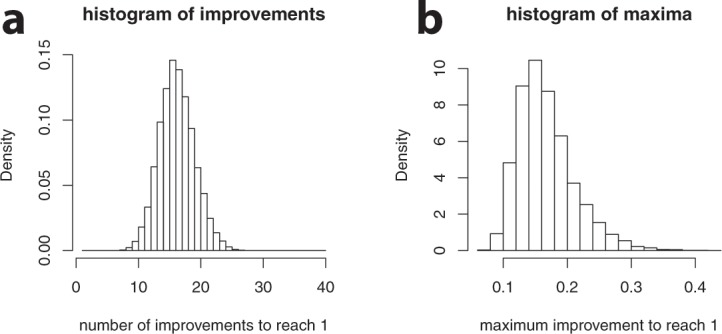
Histograms of the number of improvements needed to reach a total improvement of 1 (**a**) and the maximal step size that occurred in each run (**b**), Parameters were α = 30 and N = 1500.

The distributions of improvements can be used to estimate how many improvements are necessary to achieve a given total improvement *m* and what the maximum size of improvements was. The (mean) number of improvements necessary is not equal to *m* divided by the mean size of an improvement, but somewhat larger: because improvements come in discrete steps, the total improvement achieved will in general exceed *m* by a little bit. Therefore it is easier to simulate this with Monte-Carlo techniques. This is implemented by accumulating random improvements until they exceed the threshold *m*. For *m* = 1, *α* = 30 and *N* = 1500, the histograms of the number of improvements and the maximum size of improvements in each string of improvements are given in Fig. 6.

The following observations can be made: first, the number of necessary steps is larger than 1. Even for a modest (total) improvement (e.g. 1), it is exceedingly unlikely that an improvement of this magnitude would occur in a single step. Second, relatively large improvements (i.e. with a fitness effect between 0.1 and 0.4) are common. For almost all simulations, improvements with effects of this size occurred. This is in line with the observation by Eyre-Walker and Keightley that “[…] *large-effect mutations seem to be those that contribute most to adaptation*.”^20, p. 614^. Even though beneficial mutations of large effect are rare, they are more likely to go to fixation. This is related to our final observation, that the total number of improvements involved is generally not very large. In summary: a single mutation event is ruled out in probabilistic terms. Instead, our analysis favors an evolutionary scenario with relatively few, but relatively important events that unfold gradually (see Supplementary Material Section S6 for the precise relation between the number of mutations required and parameters *m* and *α*).

## Discussion

We have applied a variety of (standard) techniques from theoretical biology to the question of how to quantify the probability of a complex trait like language evolving in a single step, in many small steps, or in a limited number of intermediate steps, within a specific time window and population size. Our model estimates the probability of a mutation that improves fitness with a given amount using extreme value theory^8^ and then uses diffusion theory to estimate the probability^10^ and the time^19^ it takes for such mutations to go to fixation. Finally it uses Monte Carlo simulation to estimate how many mutations are needed to reach a given gain in fitness and how large these mutations are. The dynamics of the model we studied shows that a limited number of intermediate steps is most probable. On these grounds we conclude that the single-mutant hypothesis is not independently motivated by evolutionary dynamics.

Even though our model is valid for the evolution of any trait, our investigation revolves around language because of the existing literature and specifically because of the influential and detailed hypothesis formulated most recently by Berwick and Chomsky^3^. Our conclusion challenges a key Chomskyan argument in favor of Merge and the strongest minimalist hypothesis^3^, because we have shown that it is not correct to argue that fixation of multiple mutations is less probable than fixation of a single macro-mutation. By questioning the proposed evolutionary rationale for a single, un-decomposable computational innovation, we challenge one of the central theory-external motivations for Merge. Although our scenario allows the possibility of an ability for Merge that is based on an accumulation of a larger number of mutations, this is not in line with Berwick and Chomsky’s view of Merge as an atomic ability^3^, i.e. an ability one either has or does not have. For complementary challenges to more internal motivations for Merge, see Martins and Boeckx^4^.

Combined with mounting, compelling evidence from the archeological record that goes against a very recent ‘great leap forward’^22,23^, against the very possibility of formulating a single origin of modern humans^23^ and that indicates that the ability for language is older and evolved over a longer time than hitherto thought^24–26^, as well as genetic evidence that shows the rarity of truly fixed mutations in the modern human lineage^27–29^ (none of them specifically tied to the language phenotype), we are inclined to argue that evidence favors the view that language emerged through a gradual accumulation of mutations. Moreover, analysis of the evolutionary process of culturally transmitted communicative abilities^30–32^ indicates that one needs to take into account the co-evolution of genes and culture. We therefore propose that gene-culture co-evolution is a more compelling general approach to the human language faculty.

## Supplementary information

Supplementary Information.
