# Supplementary material for: Evolutionary Dynamics Do Not Motivate a Single-Mutant Theory of Human Language
Source: Sci Rep. 2020 Jan 16;10:451. doi: 10.1038/s41598-019-57235-8 (PMC6965110; doi:10.1038/s41598-019-57235-8)
Supplement: Supplementary file 1 — Supplementary Information. [file 41598_2019_57235_MOESM1_ESM.docx]

Supplementary Information for

**Evolutionary Dynamics Do Not Motivate a Single-Mutant Theory of Human Language**

**Authors:**

Bart de Boer^1^

Bill Thompson^2^

Andrea Ravignani^1,3^

Cedric Boeckx^4,5,6^

**Affiliations:**

1. AI-lab, Vrije Universiteit Brussel, Pleinlaan 2, 1050 Brussel, Belgium

2. Language and Cognition Department, Max Planck Institut für Psycholinguistik, Wundtlaan 1, 6525 XD Nijmegen, the Netherlands

3. Research Department, Sealcentre Pieterburen, Hoofdstraat 94a, 9968 AG Pieterburen, The Netherlands

4. ICREA, Passeig Lluís Companys 23, 08010 Barcelona, Spain

5. Institute for Complex Systems, Universitat de Barcelona, 585 Gran Via, 08007 Barcelona, Spain

6. Section of General Linguistics, Universitat de Barcelona, 585 Gran Via, 08007 Barcelona, Spain

**Corresponding Author:**

Bart de Boer

AI-lab, Vrije Universiteit Brussel

Pleinlaan 2

1050 Brussel

Belgium

+32 2 629 3755

bart@ai.vub.ac.be

**This PDF file includes:**

Supplementary text

References for SI citations

# S0. General approach

The diffusion approach only considers the evolution of the proportion *p* of mutant alleles *A* (while the wild-type allele is denoted *a*). Following Kimura^1^, a finite population of diploid individuals can be approximated with a diffusion model based on the following backward Kolmogorov equation^1, eq. 1^:

${\frac{\partial u\left( p,t \right)}{\partial t}=M}_{\delta p}\left( p \right)\frac{\partial u\left( p,t \right)}{\partial p}+{\frac{1}{2}V}_{\delta p}\left( p \right)\frac{\partial^{2}u\left( p,t \right)}{\partial p^{2}}$

where $u\left( p,t \right)$ is the probability of the mutant allele becoming fixed at time *t*, if the population starts with a proportion of *p* at time *t*= 0 (where the unit of *t* is generations). $M_{\delta p}\left( p \right)$ is the mean change in proportion *p* per generation, and $V_{\delta p}\left( p \right)$ its variance.

In order to know the probability of fixation the following ordinary differential equation needs to be solved:

${0=M}_{\delta p}\left( p \right)\frac{\partial u\left( p,t \right)}{\partial p}+{\frac{1}{2}V}_{\delta p}\left( p \right)\frac{\partial^{2}u\left( p,t \right)}{\partial p^{2}}$

Calculating the probability of the mutant taking over the population requires the boundary conditions:

$\begin{matrix} u\left( 0,\infty\right)=0 \\ u\left( 1,\infty\right)=1 \end{matrix}$

i.e. if the population starts without mutants, the probability of them taking over is zero, and if the population starts with all mutants, they have already taken over, so the probability of takeover is 1.

# S1. Mean and Variance Approximations

In this section we provide three ways of formulating mean and variance in the backwards Kolmogorov equation and compare them with a direct Monte-Carlo simulation of the selection process.

The first models the population as if it were haploid. The mean change and its variance are then:

$M_{\delta p}\left( p \right)=p\left( 1-p \right)\frac{s_{aA}}{1+s_{aA}\cdot p}$

$V_{\delta p}\left( p \right)=\frac{p\left( 1-p \right)}{2N}\frac{2+s_{aA}}{1+s_{aA}\cdot p}$

where *s_aA_* is the selection coefficient of the heterozygote mutant (i.e. assuming the fitness of the wild type is 1, the fitness of the heterozygote mutant is 1+*s_aA_*) and *N* is the population size.

A more precise approximation also considers the influence of the homozygote mutants. This leads to the following expressions:

$M_{\delta p}=p\left( 1-p \right)\frac{s_{aA}+\left( s_{AA}-2s_{aA} \right)p}{\bar{w}}$

$V_{\delta p}=\frac{p\left( 1-p \right)}{2N}\cdot\frac{\left( 1+s_{aA}+\left( s_{AA}-s_{aA} \right)p \right)\left( 1+s_{aA}\cdot p \right)}{\bar{w}^{2}}$

Where:

$\bar{w}=1+2s_{aA}p\left( 1-p \right)+s_{AA}p^{2}$

is the mean fitness of the population, and *s_AA_* is the selection coefficient of heterozygote mutants.

A third model is due to Kimura ^1^ in which it is assumed that the selection coefficients are small, such that we can ignore the effect of the mean fitness. Also, Kimura assumes the use of the effective population size, such that the following expressions are obtained:

$M_{\delta p}\left( p \right)=p\left( 1-p \right)\left( s_{aA}+\left( s_{AA}-2s_{aA} \right)p \right)$

$V_{\delta p}\left( p \right)=\frac{p\left( 1-p \right)}{2N_{e}}$

where *N_e_* is the *effective population size*, i.e. the size of a randomly mating population that behaves the same as the actual population (which may not have random mating).

Table 1: Comparison of fixation probabilities of the different models with a direct simulation for different values of N (rows) and the selection coefficient (columns). For the simulation, the 95% confidence interval is given in brackets.

|  |  | **0.1** | **0.3** | **1.0** |
| --- | --- | --- | --- | --- |
| **100** | *Precise* | 0.156 | 0.363 | 0.632 |
|  | *Kimura* | 0.171 | 0.443 | 0.862 |
|  | *Haploid* | 0.173 | 0.407 | 0.736 |
|  | *MC-simulation* | 0.171 (0.163, 0.178) | 0.413 (0.404, 0.423) | 0.794 (0.786,0.801) |
| **300** | *Precise* | 0.162 | 0.368 | 0.632 |
|  | *Kimura* | 0.178 | 0.448 | 0.863 |
|  | *Haploid* | 0.173 | 0.407 | 0.736 |
|  | *MC-simulation* | 0.173 (0.166, 0.180) | 0.417 (0.409, 0.427) | 0.781 (0.774, 0.790) |
| **1000** | *Precise* | 0.164 | 0.368 | 0.631 |
|  | *Kimura* | 0.179 | 0.449 | 0.864 |
|  | *Haploid* | 0.173 | 0.407 | 0.736 |
|  | *MC-simulation* | 0.179 (0.171, 0.187) | 0.419 (0.411, 0.428) | 0.797 (0.788, 0.805) |

### Quality of the Approximations

In order to compare the quality of the different approximations, they were compared to a direct simulation of the population. This simulation modeled a population that is repeatedly replaced by a new population in which the new individuals descend from two individuals in the previous population, each of which was selected with a probability that was proportional to their fitness. The fitness was 1 for the wild type, 1+*s_aA_* for the heterozygote mutant and 1+*s_AA_* for the homozygote mutant.

The fixation probabilities are presented in Table 1. It shows them for three different population sizes (100, 300 and 1000) and three different selection coefficients (0.1, 0.3 and 1.0) ­that were equal for homozygotes and heterozygotes (*s_aA_* = *s_AA_*) – as was pointed out in section 2.1, this corresponds to the mutation being dominant, and this appears the most likely interpretation for Berwick and Chomsky’s single-mutant hypothesis. It is clear from these results that none of the models is entirely accurate, that accuracy increases with decreasing selection coefficient and increasing population (as expected), and that the simplest approximation (assuming a haploid population) works surprisingly well. Larger population sizes were not simulated, because they were impractical, and because they were not expected to change the conclusions (accuracy increases with population size, so differences between models would become less pronounced). Smaller selection coefficients were also not simulated, because all models have good accuracy for small selection coefficients. As was pointed out in section 2.1, higher selection coefficients were considered extremely unlikely.

# S2. Diffusion Approximation of the Moran Process

The equations that are used for investigating fixation probabilities for the haploid approximation (equations 1.4 and 1.5) in the main text are derived from the Moran process. The Moran process ^2,3, ch. 6^ is a birth-death process in a finite population of size *M* where at each time step, one member of the population dies, and one new member of the population gets born. The population consists of residents and mutants, and its state can be fully described by a single integer, the number of mutants: 0 ≤ *i* ≤ *N*. At each birth death event, the number of mutants may stay the same, increase by one or decrease by one. If residents have fitness 1 and mutants have fitness 1+*s_aA_*, then the probabilities of these transitions are as follows:

$p\left( i+1 | i \right)=\frac{M-i}{M}\cdot\frac{i\left( 1+s_{aA} \right)}{M+i\cdot s_{aA}}=\frac{M-i}{M}\cdot\frac{i}{M}\cdot\frac{1+s_{aA}}{1+\frac{i}{M}s_{aA}}$

and:

$p\left( i-1 | i \right)=\frac{i}{M}\cdot\frac{M-i}{M+i\cdot s_{aA}}=\frac{i}{M}\cdot\frac{M-i}{M}\cdot\frac{1}{1+\frac{i}{M}s_{aA}}$

where in both cases the first formulation illustrates that the probability is just the product of one type dying and the other being born (taking into account the fitness) and the second formulation is a more convenient form for further analysis.

By making the substitution $p\equiv i/M$ (*i.e. p* represents the proportion of mutants), the equations can be rewritten to:

$p\left( p+\frac{1}{M} | p \right)=p\left( 1-p \right)\cdot\frac{1+s_{aA}}{1+p\cdot s_{aA}}$

$p\left( p-\frac{1}{M} | p \right)=p\left( 1-p \right)\cdot\frac{1}{1+p\cdot s_{aA}}$

These can be incorporated in the mean and variance terms of the backward Kolmogorov equation. By definition, they are as follows:

$M_{\delta p}\left( p \right)=\int\delta p\cdot w\left( p+\delta p | p \right)d\left( \delta p \right)$

$V_{\delta p}\left( p \right)=\int\left( \delta p \right)^{2}\cdot w\left( p+\delta p | p \right)d\left( \delta p \right)$

where $w\left( p+\delta p | p \right)$ is the probability per unit time to go from a state with p mutants to one with *p*+*δp* mutants. For a one-step process (i.e. a process where composition of the underlying discrete population changes by maximally one at each time step) like the Moran process, *w* can be formulated as follows:

$w\left( q | p \right)=\alpha\left[ \delta\left( q-p+\frac{1}{M} \right)p\left( p-\frac{1}{M} | p \right)+\delta\left( q-p-\frac{1}{M} \right)p\left( p+\frac{1}{M} | p \right) \right]$

where *α* is a conversion constant that converts the continuous time variable of the diffusion process to the discrete time variable of the Moran process, and *δ*( *x* ) is the Dirac delta function. Substituting this in equations and gives:

$M_{\delta p}=\frac{\alpha}{M}\left[ p\left( p+\frac{1}{M} | p \right)-p\left( p-\frac{1}{M} | p \right) \right]$

$V_{\delta p}=\frac{\alpha}{M^{2}}\left[ p\left( p+\frac{1}{M} | p \right)+p\left( p-\frac{1}{M} | p \right) \right]$

These formulations are still general for any one-step process. Substituting and gives the formulation for the Moran process:

$M_{\delta p}=\frac{\alpha}{M}p\left( 1-p \right)\frac{s_{aA}}{1+p\cdot s_{aA}}$

$M_{\delta p}=\frac{\alpha}{M^{2}}p\left( 1-p \right)\frac{{2+s}_{aA}}{1+p\cdot s_{aA}}$

Choosing *α* = *M* ensures that time is expressed in generations (i.e. the number of time steps needed to replace a number of agents that is equal to the population size). Finally, because we are modeling a diploid population (admittedly pretending we can do that by only looking at the heterozygotes) we need to choose *M* = 2*N*, and we obtain equations 1.4 and 1.5 of the main text.

**S3. Frequency-dependent fitness**

When the fitness of a mutant individual is linearly dependent on the frequency *p* of mutants in the population, the equations for the selection coefficients are:

$\begin{matrix} s_{aA}\left( p \right)=\sigma_{aA}\cdot p \\ s_{AA}\left( p \right)=\sigma_{AA}\cdot p \end{matrix}$

where *s_aA_*(*p*) and *s_AA_*(*p*) are the selection coefficients of the heterozygote respectively the homozygote and *σ_aA_* and *σ_AA_* are the slope of the corresponding selection coefficients. These frequency dependent selection coefficients can be substituted in the equations for the mean change and the variance in and , and the fixation probabilities (and fixation times) can be estimated.

**S4. Probability of mutations - details**

Experimental evidence is in general agreement with the predictions implied by Orr’s model^4^ but because advantageous mutations are so rare the overall evidence is weak and could also fit other potential distributions. Eyre-Walker and Keightley also point out a potential weakness in Orr's analysis: it assumes that fitness values are drawn from a constant distribution, but in reality this distribution may be variable over time. A computational model by Cowperthwaite et al.^5^ lends support to this criticism: it shows that in their model there are many more small effect mutations, and only when "*the vast majority*" of these are ignored, does the distribution of fitness effects become exponential. It also shows that the distribution of mutant fitness depends on the parent fitness, lending support to the hypothesis that adaptation to the fitness landscape modifies the distribution of mutant fitness.

There are at least three reasons why in the analysis presented here an exponential distribution is nevertheless reasonable. The first is pointed out by Eyre-Walker and Keightley^4, p. 614^: "*Although the Gillespie–Orr prediction* [i.e. the exponential distribution of mutant fitness] *might not be correct for all advantageous mutations, it may apply to mutations of large effect. Such large-effect mutations seem to be those that contribute most to adaptation.*" This is related to the second reason: the single-mutant hypothesis proposes a mutation of very large effect, and the probability of this would lie well within the exponential region. Assuming an exponential distribution and ignoring all beneficial mutations of small effect likely overestimates the probability of the emergence of a ‘Merge mutant’, and is therefore a conservative approximation. The final reason is purely pragmatic: there is no closed form (nor even a clear empirical form) of the true distribution of mutant fitness, and the exponential distribution is relatively easy to work with.

**S5. Probability of a single improvement - derivations**

Equation (6) results in the following expression for the probability density function (with appropriate renaming of the variables, $\alpha=1/E\left[ \Delta_{1} \right]$and a scaling factor *β* to guarantee it integrates to 1):

$p\left( s \right)=\frac{\alpha}{\beta}e^{-\alpha s}\frac{1-e^{-4\frac{s}{2+s}}}{1-e^{-4N\frac{s}{2+s}}}$

This expression is somewhat unwieldy, as it does not appear that there is a closed form solution to its integral. However, observing that because *α* is large the probability drops off quickly, such that most improvements occur for small *s*, the fixation probability term can be simplified to:

$p\left( s \right)=\frac{\alpha}{\beta}e^{-\alpha s}\frac{1-e^{-2s}}{1-e^{-2Ns}}$

but although this can be integrated, it does not result in a very useful closed form solution. Replacing the second term by 1–*e*^2^*^s^* results in a good approximation for *Ns* ≫ 0, but unfortunately, it assigns zero probability to mutations with a very small selection coefficient (this should be 1/*N*). However, adding a correction term of ${e^{-\left( N+1 \right)s}}/N$ results in an excellent approximation for all *s*, resulting in our final equation for the probability of an improvement:

$p\left( s \right)=\frac{\alpha}{\beta}e^{-\alpha s}\left( 1-e^{-2s}+\frac{e^{-\left( N+1 \right)s}}{N} \right)$

which is straightforward to integrate for calculating the scaling factor *β* and the cumulative distribution function. This results in the following equations. The scaling factor is:

$\beta=\frac{2}{\alpha\left( 2+\alpha\right)}+\frac{1}{N\left( N+\alpha+1 \right)}$

**S6. Sensitivity of number of mutations to parameters *m* and α**

The relationship between the slope of the exponential distribution that describes the probability of mutations that provide a given positive selection coefficient (‒*α*), the total improvement that needs to be achieved (*I*) and the expected number of mutations (‖m‖) has been calculated through drawing random samples from the appropriate distribution (equation 15 in the main text) for different values of *α* and *I*. The mean value of the needed improvements is given in figure S3.1.

For larger numbers of mutations, this number can be approximated with satisfactory accuracy by dividing the total improvement by the mean size of a mutation that goes to fixation. This is given by:

$\left\| m \right\|\approx I\cdot\frac{\alpha\left( 2+\alpha\right)}{2+2\alpha}$

 Figure S3.1: Mean number of improvements needed to reach a given size of improvement *I* for different values of the slope the distribution of mutation sizes –*α*. The approximate equation for this relation is given in the top left corner of the graph.

**S7. The role of epistasis**

The effects of epistasis would be limited. First of all the mathematical analysis shows that genes go to fixation relatively quickly, *if* they go to fixation (counterintuitively, this is even true for very small fitness effects). This means that most of the time, only one mutation will be fixating in the population. A second mutation in the population can occur in two ways: 1) In an individual that already has the other mutation, or 2) in an individual that does not have the other mutation.

Case 1) is simple, as our mutation model only consider mutations that improve fitness, and therefore by assumption the new mutation would add to the fitness of the mutation already present (and thus would have an additive effect with respect to individuals that do not have either of the two mutations).

Case 2) is more complicated, as what happens depends on the fitness effects of the two mutations separately and when they occur in the same individual. By definition our probability model considers the combination of the mutation probability and the probability of it going to fixation (if it would occur in isolation). We can therefore assume that we are only considering situations where both mutations become at least frequent enough that individuals that have both mutations become frequent as well. There are then two new possibilities:

2.1) The individual with both mutations has higher fitness than either of the other types in the population or 2.2) The individual has lower fitness.

In case 2.1, we expect a high probability that the double mutants take over the population: the single mutants are already well-established, so new double mutants are formed frequently, and as these have higher fitness they are expected to spread. Because double mutants are formed so frequently, we expect the spread to happen at least as quickly as ordinary single mutants.

In case 2.2, individuals combining the two mutations will have a disadvantage, so we expect that the single mutation with the highest fitness effect will eventually become dominant.

Only case 2.2 may result in a slightly slower evolution of the complex trait.

**References**

1. Kimura, M. Average time until fixation of a mutant allele in a finite population under continued mutation pressure: Studies by analytical, numerical, and pseudo-sampling methods. *Proc. Natl. Acad. Sci.* **77**, 522–526 (1980).

2. Moran, P. A. P. Random Processes in Genetics. *Math. Proc. Camb. Philos. Soc.* **54**, 60–71 (1958).

3. Nowak, M. A. *Evolutionary Dynamics*. (Harvard University Press, 2006).

4. Eyre-Walker, A. & Keightley, P. D. The distribution of fitness effects of new mutations. *Nat. Rev. Genet.* **8**, 610 (2007).

5. Cowperthwaite, M. C., Bull, J. J. & Meyers, L. A. Distributions of Beneficial Fitness Effects in RNA. *Genetics* **170**, 1449 (2005).
